# The complete genomes of three viruses assembled from shotgun libraries of marine RNA virus communities

**DOI:** 10.1186/1743-422X-4-69

**Published:** 2007-07-06

**Authors:** Alexander I Culley, Andrew S Lang, Curtis A Suttle

**Affiliations:** 1 University of British Columbia, Department of Botany, 3529-6270 University Blvd, Vancouver, B.C. V6T 1Z4, Canada; 2 Department of Biology, Memorial University of Newfoundland, St. John's, NL A1B 3X9, Canada; 3 University of British Columbia, Department of Earth and Ocean Sciences, Department of Microbiology and Immunology, 1461-6270 University Blvd, Vancouver, BC, V6T 1Z4, Canada

## Abstract

**Background:**

RNA viruses have been isolated that infect marine organisms ranging from bacteria to whales, but little is known about the composition and population structure of the *in situ *marine RNA virus community. In a recent study, the majority of three genomes of previously unknown positive-sense single-stranded (ss) RNA viruses were assembled from reverse-transcribed whole-genome shotgun libraries. The present contribution comparatively analyzes these genomes with respect to representative viruses from established viral taxa.

**Results:**

Two of the genomes (JP-A and JP-B), appear to be polycistronic viruses in the proposed order *Picornavirales *that fall into a well-supported clade of marine picorna-like viruses, the characterized members of which all infect marine protists. A temporal and geographic survey indicates that the JP genomes are persistent and widespread in British Columbia waters. The third genome, SOG, encodes a putative RNA-dependent RNA polymerase (RdRp) that is related to the RdRp of viruses in the family *Tombusviridae*, but the remaining SOG sequence has no significant similarity to any sequences in the NCBI database.

**Conclusion:**

The complete genomes of these viruses permitted analyses that resulted in a more comprehensive comparison of these pathogens with established taxa. For example, in concordance with phylogenies based on the RdRp, our results support a close homology between JP-A and JP-B and RsRNAV. In contrast, although classification of the SOG genome based on the RdRp places SOG within the *Tombusviridae*, SOG lacks a capsid and movement protein conserved within this family and SOG is thus likely more distantly related to the *Tombusivridae *than the RdRp phylogeney indicates.

## Background

RNA viruses of every classification have been isolated from the ocean; nevertheless, the marine RNA virus community remains largely uncharacterized. Although there are several examples of RNA viruses that infect marine animals [1] these organisms represent a very small portion of the organisms in the sea; therefore it is unlikely that viruses infecting these organisms make up a significant fraction of the natural RNA virioplankton. Marine RNA phages appear to be rare [2] and thus it is more likely that the dominant RNA viruses infect the diverse and abundant marine protists. For example, RNA viruses have recently been isolated that infect a number of marine protists including a diatom [3], a dinoflagellate [4], a raphidophyte [5], a prasinophyte [6] and a thraustochytrid [7].

Picorna-like viruses are a "superfamily" of positive-sense single-stranded RNA (ssRNA) viruses that have similar genome features and several conserved protein domains [8]. Previously, we investigated the diversity of marine picorna-like viruses by analysis of RNA-dependent RNA polymerase (RdRp) sequences amplified from marine virus communities and demonstrated that picorna-like viruses are present and persistent in a diversity of marine environments [9]. Furthermore, phylogenetic analyses showed that none of the environmental sequences fell within established virus families.

In a recent study, reverse-transcribed whole-genome shotgun libraries were used to characterize two marine RNA virus communities [10]. Positive-sense ssRNA viruses that are distant relatives of known RNA viruses dominated the libraries. One RNA virus library (JP) was characterized by a diverse, monophyletic clade of picorna-like viruses, but the second library (SOG) was dominated by viruses distantly related to members of the family *Tombusviridae *and the genus *Umbravirus*. Moreover, in both libraries, a high percentage of sequence fragments were part of only a few contiguous segments of sequence (contigs). Specifically, in the SOG sample 59% of the sequence fragments formed a single contig. Similarly, 66% of JP sequence fragments contributed to only four contigs that represented two viral genomes. Using a RT-PCR-based approach to increase the amount of sequence for each dominant contig resulted in the assembly of three complete viral genomes. This contribution analyzes these genomes from three previously unknown marine RNA viruses and investigates their similarities and differences with respect to representative genotypes from established viral taxa.

## Results and Discussion

### Jericho Pier site

**Figure 1:**
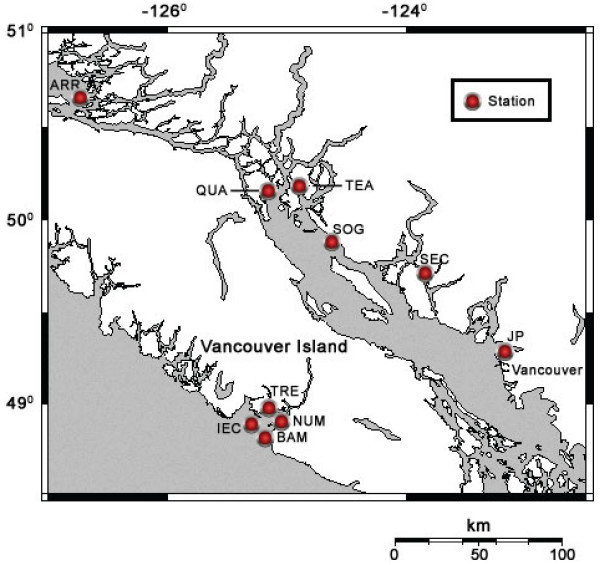
**Map of southwestern British Columbia, Canada showing locations where samples were collected.**Sites in coastal BC waters where the JP-A and JP-B genomes were detected are indicated and labelled. Both JP-A and JP-B were detected in samples from 5 of the 9 stations that were screened. The SOG station was not assayed for JP-A or JP-B. See Table 2 for additional information about the stations.

The two assembled genomes (JP-A and JP-B) from the Jericho Pier sampling site (Figure 1) are single molecules of linear ssRNA.

**Figure 2:**
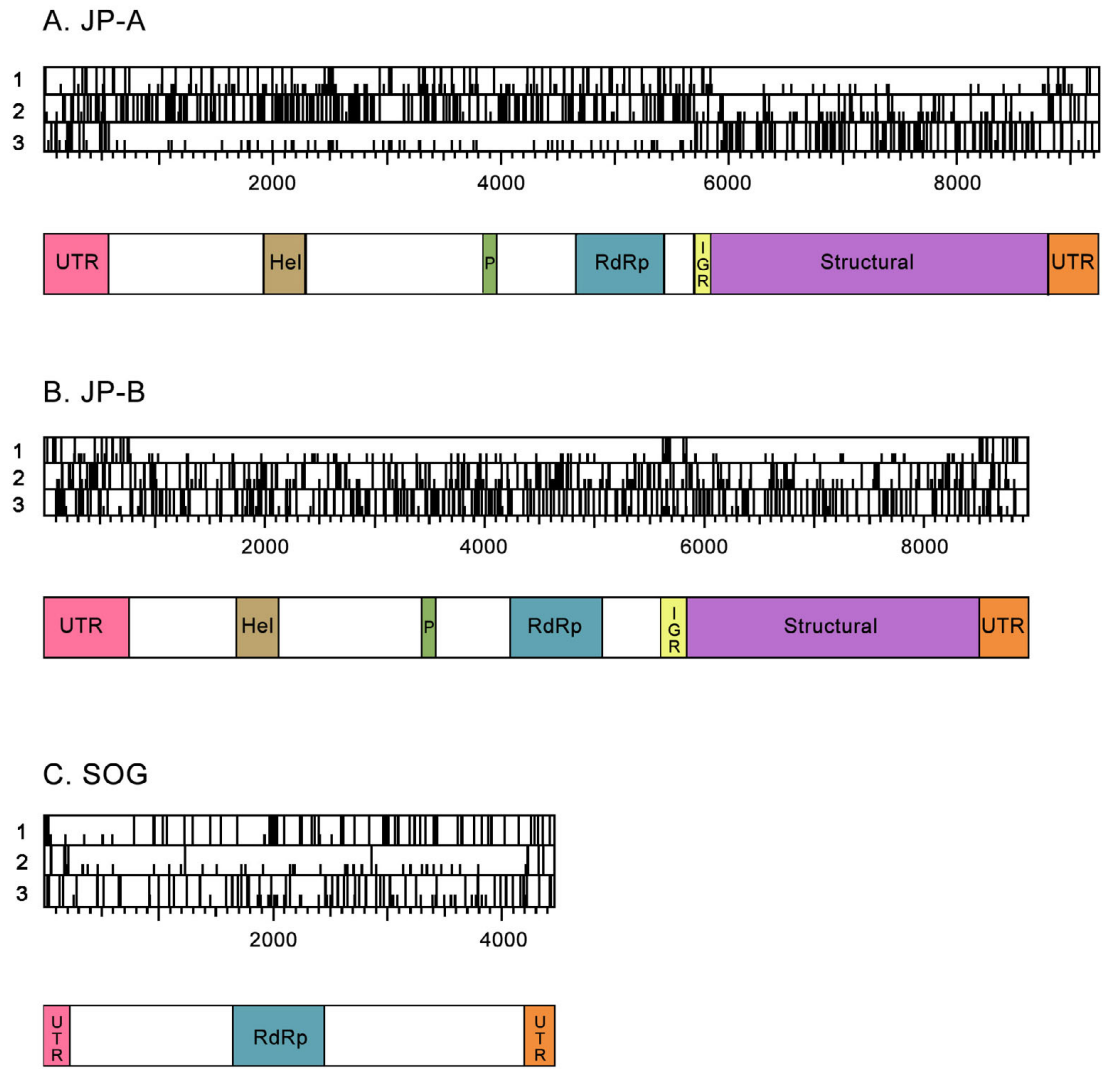
Analysis of genomes for putative open reading frames. In the ORF maps created with DNA Strider [28], for each reading frame, potential start codons (AUG) are shown with a half-height line and stop codons (UGA, UAA, and UAG) are shown by full-height lines. Recognizable conserved RNA virus protein domains (Hel = helicase, Pro = Protease, RdRp = RNA-dependent RNA polymerase) and other genomic features (UTR = untranslated region, IGR = intergenic region) are noted below each genome. See text for more detail. A. Map of the JP-A genome. B. Map of the JP-B genome. C. Map of the SOG genome.

The JP-A genome is positive-sense, 9212 nt in length with a 632 nt 5' untranslated region (UTR) followed by 2 predicted open reading frames (ORFs) of 5067 nt (ORF 1, nt position 633 to 5699) and 3044 nt (ORF 2, nt position 5848 to 8799) separated by an intergenic region (IGR) of 149 nt (Figure 2A). ORF 2 is followed by a 3' UTR of 413 nt (nt position 8800 to 9212) and a polyadenylate [poly (A)] tail. The base composition of JP-A is 27.1% A, 19.4% C, 22.0% G, and 31.6% U; this results in a G+C of 41%, a percentage similar to other polycistronic picorna-like viruses (Table 1).

**Table 1:** Comparison of base composition between polycistronic picorna-like viruses

Genome*	A	C	G	U	% G+C
**JP-A**	**27.1**	**19.4**	**22.0**	**31.6**	**41**
**JP-B**	**30.8**	**17.9**	**19.7**	**31.6**	**38**
ABPV	35.7	15.4	20.1	28.9	36
ALPV	31.3	19.4	19.2	30.2	39
BQCV	29.2	18.5	21.6	30.6	40
CrPV	32.6	18.4	20.9	28.1	39
DCV	29.9	16.3	20.4	33.4	37
HiPV	29.2	18.7	20.9	31.2	39
KBV	33.8	17.5	20.2	28.6	38
PSIV	31.3	17.0	19.4	32.3	36
RhPV	30.0	18.6	20.2	31.2	39
RsRNAV	31.2	16.7	19.5	32.5	36
SINV-1	32.9	18.3	20.5	28.2	39
SssRNAV	24.2	26.1	23.6	26.0	50
TSV	28.0	20.2	23.0	28.8	43
TrV	28.7	16.1	19.8	35.4	36
					
Average	30.4	18.4	20.7	30.5	39

* See Additional file 2 for the complete virus names

Comparison to known viral sequences shows that the protein sequence predicted to be encoded by ORF 1 of JP-A contains conserved sequence motifs characteristic of a type III viral Helicase (aa residues 430 to 545), a 3C-like cysteine protease (aa residues 1077 to 1103) and a type I RdRp (aa residues 1350 to 1591) [11] (Figure 1A). BLASTp [12] searches of the NCBI database with the predicted ORF 1 protein sequence showed significant sequence similarities (*E *value < 0.001) to nonstructural protein motifs of several viruses, including members of the families *Dicistroviridae *(Drosophila C virus), *Marnaviridae *(HaRNAV)*, Comoviridae *(Cowpea mosaic virus) and the unassigned genus *Iflavirus *(Kakugo virus). The top matches for ORF 1 were to RsRNAV [*E *value = 3 × 10^-119^, identities = 302/908 (33%)], a newly sequenced, unclassified positive-sense ssRNA virus that infects the widely distributed diatom *Rhizosolenia setigera *[3], HaRNAV [*E *value = 2 × 10^-32^, identities = 156/624 (25%)] and Drosophila C virus [*E *value = 1 × 10^-29^, identities = 148/603 (24%)], a positive-sense ssRNA virus that infects fruit flies. Comparison of the protein sequence predicted to be encoded by ORF 2 of JP-A to known viral sequences shows that it has significant similarities to the structural proteins of viruses from the families *Dicistroviridae *(Drosophila C virus), *Marnaviridae *(HaRNAV), and the genus *Iflavirus *(*Varroa destructor *virus 1). The sequences that are most similar to ORF 2 of JP-A were the structural protein regions of RsRNAV [*E *value = 6 × 10^-78^, identities = 212/632 (33%)], HaRNAV [*E *value = 6 × 10^-68^, identities = 187/607 (30%)] and SssRNAV [*E *value = 2 × 10^-49^, identities = 241/962 (25%)].

The JP-B RNA genome is also likely from a positive-sense ssRNA virus. The 8839 nt genome consists of a 5' UTR of 774 nt followed by two predicted ORFs of 4842 nt (ORF 1, nt position 775 to 5616) and 2589 nt (ORF 2, nt position 5914 to 8502) separated by an IGR of 298 nt (nt position 5617 to 5913) (Figure 2B). The 3' UTR is 337 nt long and followed by a poly (A) tail. The base composition of the genome is A, 30.8%; C, 17.9%; G, 19.7%; U, 31.6%. Like JP-A, this % G+C value of 38% is comparable to the % G+C observed in other polycistronic picorna-like viruses (Table 1).

The position of core sequence motifs conserved among positive-sense ssRNA viruses and BLAST searches of the NCBI database with the translated JP-B genome suggest that nonstructural proteins are encoded by ORF1, and the structural proteins are encoded by ORF2. We identified conserved sequence motifs in ORF 1 characteristic of a type III viral Helicase (aa residues 328 to 441), a 3C-like cysteine protease (aa residues 882 to 909) and a type I RdRp (aa residues 1143 to 1408) [11] (Figure 2B). BLASTp [12] searches of the GenBank database showed that ORF 1 has significant similarities (*E *value < 0.001) to nonstructural genes from positive-sense ssRNA viruses from a variety of families, including the *Comoviridae *(Peach rosette mosaic virus), *Dicistroviridae *(Taura syndrome virus), *Marnaviridae *(HaRNAV)*, Sequiviridae *(Rice tungro spherical virus) and *Picornaviridae *(Avian encephalomyelitis virus). The top scoring sequences [*E *value = 2 × 10^-69^, identities = 232/854 (27%)] were to a RdRp sequence from RsRNAV and a partial picorna-like virus RdRp from an unidentified virus [*E *value = 2 × 10^-40^, identities = 85/150 (56%)] amplified from the same JP station during an earlier study [9]. Significant similarities to ORF 2 include the structural genes of viruses from the families *Dicistroviridae *(*Rhopalosiphum padi *virus), *Marnaviridae *(HaRNAV) and *Picornaviridae *(Human parechovirus 2), as well as the unclassified genus *Iflavirus *(*Ectropis obliqua *picorna-like virus). The top scoring sequences were to the capsid protein precursor regions of RsRNAV [*E *value = 9 × 10^-88^, identities = 244/799 (30%)] and HaRNAV [*E *value = 8 × 10^-60^, identities = 180/736(24%)] and SssRNAV [*E *value = 1 × 10^-40^. identities = 156/588 (26%)].

The JP-A and JP-B genomes appear to have a polycistronic genome organization similar to that found in viruses in the family *Dicistroviridae*. Several of these viruses contain internal ribosome entry sites (IRES) [13-16] that position the ribosome on the genome, actuating translation initiation even in the absence of known canonical initiation factors [13]. For example, TSV, a marine dicistrovirus, has an IRES located in the IGR that directs the synthesis of the structural proteins [15]. Computational searches did not identify the secondary structure elements characteristic of dicistrovirus IGR-IRESs in the JP genomes [16,17], however, JP-A and JP-B genomes have extensive predicted secondary structure in the 5' UTRs and IGRs [18,19], suggestive of an IRES function. Moreover, start codons in a favorable Kozak context, i.e. conserved sequences upstream of the start codon that are thought to play a role in initiation of translation [20], were not found in the JP genomes. However to unequivocally demonstrate IRES elements in the JP genomes, they must be confirmed experimentally in polycistronic constructs. Nevertheless, it seems reasonable that JP-A and JP-B use similar mechanisms to initiate translation of the ORF 2 genes as are known to be employed by several dicistroviruses.

We used RT-PCR to assess the distribution and persistence of the JP-A and JP-B viruses *in situ*. Amplification with specific primers that target each of these viruses occurred in samples from throughout the Strait of Georgia, the West coast of Vancouver Island, and in every season and tidal state at Jericho pier (Figure 1, Table 2). These results suggest that JP-A and JP-B are ubiquitous in the coastal waters of British Columbia.

**Table 2:** JP genome survey sample sites and results of assays

Station Name	Station location (B.C., Canada)	Date (mm/dd/yy)	Location (Lat., Long.)	Depth (m)	Temp (°C)	Salinity (ppt)	JP-A PCR	JP-B PCR
JP	Jericho Pier	04/28/00	49.27, -123.20	S	9	26	**+**	**+**
JP	Jericho Pier	06/15/00	49.27, -123.20	S	14	12	**+**	**+**
JP	Jericho Pier	06/29/00	49.27, -123.20	S	17	12	**+**	**+**
JP	Jericho Pier	07/06/00	49.27, -123.20	S	16	13	**+**	**+**
JP	Jericho Pier	07/13/00	49.27, -123.20	S	18	8	**-**	**-**
JP	Jericho Pier	07/27/00	49.27, -123.20	S	18	11	**+**	**+**
JP	Jericho Pier	08/17/00	49.27, -123.20	S	18	18	**+**	**+**
JP	Jericho Pier	09/14/00	49.27, -123.20	S	15	19	**+**	**+**
JP	Jericho Pier	09/21/00	49.27, -123.20	S	15	16	**-**	**+**
JP	Jericho Pier	09/28/00	49.27, -123.20	S	14	21	**+**	**+**
JP	Jericho Pier	11/23/00	49.27, -123.20	S	8	27	**+**	**+**
JP	Jericho Pier	02/15/01	49.27, -123.20	S	7	27	**+**	**+**
JP	Jericho Pier	06/14/01	49.27, -123.20	S	15	13	**+**	**+**
SEC	Sechelt Inlet	07/06/03	49.69, -123.84	4	13	26	**-**	**+**
TEA	Teakearne Inlet	07/07/03	50.19, -124.85	5	13	28	**+**	**-**
QUA	Quadra Island	07/07/03	50.19, -125.14	3	13	28	**-**	**-**
ARR	Arrow Pass	07/09/03	50.72, -126.67	2	10	31	**+**	**+**
IEC	Imperial Eagle Channel	06/20/99	48.87, -125.21	7	n.a.	n.a.	**+**	**-**
TRE	Trevor Channel	06/28/99	48.97, -125.16	S	n.a.	n.a.	**+**	**+**
BAM	Bamfield Inlet	07/06/99	48.81, -125.16	S	n.a.	n.a.	**+**	**+**
NUM	Numukamis Bay	07/12/99	48.90, -125.01	8	n.a.	n.a.	**+**	**+**

A "+" indicates amplification and "-" indicates no amplification occurred. "n.a." indicates the data is not available and "S" means the sample was taken from the surface.

It has long been recognized that several other groups of small, positive-sense ssRNA viruses share many characteristics with viruses in the family *Picornaviridae*. Recently, Christian et al. [8] proposed creating an order (the *Picornavirales*) of virus families (*Picornaviridae*, *Dicistroviridae*, *Marnaviridae*, *Sequiviridae *and *Comoviridae*) and unassigned genera (*Iflavirus*, *Cheravirus*, and *Sadwavirus*) that have picornavirus-like characteristics. Viruses in the proposed order have genomes with a protein covalently attached to the 5' end, a 3' poly (A) tail, a conserved order of non-structural proteins (Helicase-VpG-Proteinase-RdRp), regions of high sequence similarity in the helicase, proteinase and RdRp, post translational protein processing during replication, an icosahedral capsid with a unique "pseudo-T3" symmetry, and only infect eukaryotes.

**Figure 3:**
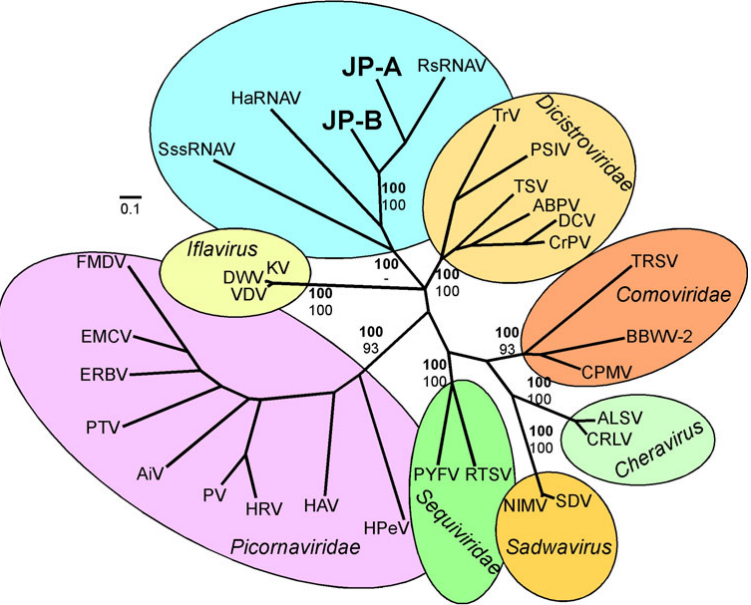
**Bayesian maximum likelihood trees of aligned RdRp amino acid sequences from the JP-A and JP-B genomes and representative members of the proposed order *Picornavirales***. Bayesian clade credibility values are shown for relevant nodes in boldface followed by bootstrap values based on neighbour-joining analysis. The Bayesian scale bar indicates a distance of 0.1. See Additional file 2 for complete virus names and accession numbers.

**Figure 4:**
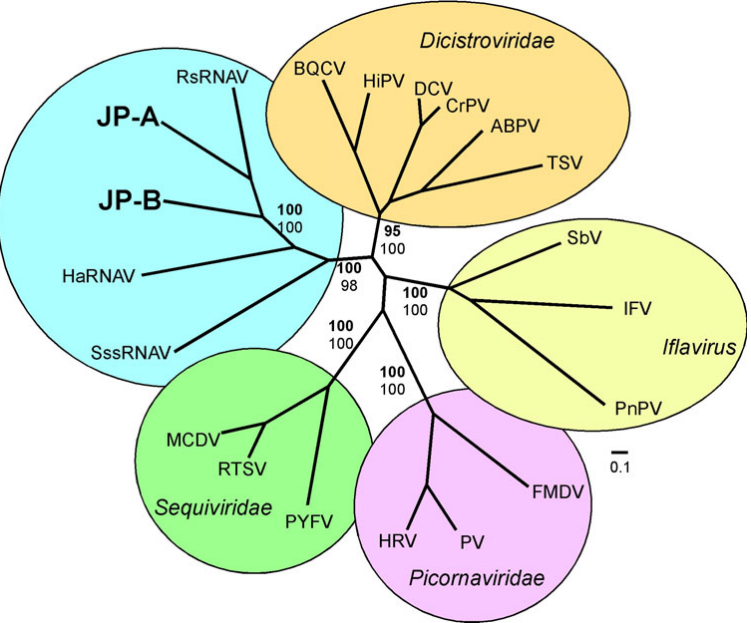
**Bayesian maximum likelihood trees of aligned concatenated helicase, RdRp and VP3-like capsid amino acid sequences from the JP-A and JP-B genomes and other picorna-like viruses**. Bayesian clade credibility values are shown for relevant nodes in boldface followed by bootstrap values based on neighbour-joining analysis. The Bayesian scale bar indicates a distance of 0.1. See Additional file 2 for complete virus names and accession numbers.

Although the capsid morphology, presence of a 5' terminal protein and replication strategy and hosts are unknown, signature genomic features and phylogenetic analyses suggest that the JP viruses fall within the proposed order *Picornavirales*. Both JP genomes encode the conserved core aa motifs and have the non-structural gene order characteristic of viruses in the proposed *Picornavirales*. Furthermore, both JP genomes have a poly (A) tail and G+C content commensurate with these other viruses. Bayesian trees [21] based on alignments of conserved RdRp domains [11] (Figure 3), as well as concatenated (putative) Hel+RdRp+VP3 capsid-like protein sequences (Figure 4), of the JP genomes and representative members of the proposed *Picornavirales*, resolves established taxa according to previous taxonomic divisions. These analyses also provide strong support for a clade comprised of viruses (HaRNAV, RsRNAV and SssRNAV) that infect marine protists and the JP-A and JP-B viruses. Within this clade, RsRNAV, JP-A and JP-B have the most characteristics in common. For example, they have the same order of structural and non-structural genes, they are polycistronic and the phylogenetic analyses indicate they are more closely related (Figures 3 and 4). Whether JP-A and JP-B infect host organisms related to *Rhizosolenia setigera *remains unclear, although because of the inclusion of the JP genomes within this clade and the fact that protists are the most abundant eukaryotes in the sea, we suggest that both JP viruses likely have a protist host.

### Strait of Georgia site

The SOG genome was assembled from the Strait of Georgia metagenomic library, and subsequently completed as described in Methods. The genome has features characteristic of a positive-sense ssRNA virus. The genome is 4449 nt long and comprised of a 5' UTR of 334 bp followed by three putative ORFs (nt position 335–1228, nt position 1385–2860 and nt position 2903–4228) and is terminated with a 3' UTR of 221 nt. A poly (A) tail was not detected. Another putative ORF located at nt position 49 to 783 is in an alternative reading frame relative to the ORFs discussed above (Figure 2C). The G+C content of the SOG genome is 52%.

We identified the eight conserved motifs of the RdRp [11] in the SOG genome (aa residues pos 451 to 700) (Figure 2C). tBLASTx [12] searches with the remainder of the genome sequence showed no significant matches (*E *value < 0.001) to sequences in the NCBI database (including the five environmental metagenomes that have been deposited). BLASTp searches with the putative RdRp sequence resulted in significant similarities (*E *value < 0.001) to RdRp sequences from positive-sense ssRNA viruses from the family *Tombusviridae *and the unassigned genus *Umbravirus*. The sequence with the most similarity to SOG was from Olive latent virus 1 [*E *value = 3 × 10^-66^, identities = 180/508 (35%)]. This virus belongs to the genus *Necrovirus *in the family *Tombusviridae *that has a host range restricted to higher plants [22]. SOG is also significantly similar to the Carrot mottle mimic virus sequence [*E *value = 6 × 10^-66^, identities = 178/492 (36%)], a member of the unclassified genus *Umbravirus *whose known members infect only flowering plants [23].

**Figure 5:**
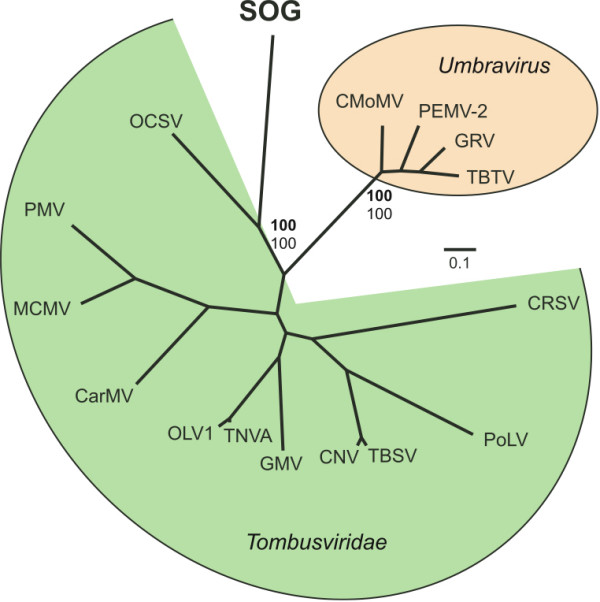
**Bayesian maximum likelihood trees of aligned RdRp amino acid sequences from the SOG genome and members of the family Tombusviridae and unassigned genus Umbravirus**. Bayesian clade credibility values are shown for relevant nodes in boldface followed by bootstrap values based on neighbour-joining analysis. The Bayesian scale bar indicates a distance of 0.1. See Additional file 2 for complete virus names and accession numbers.

Although the SOG putative RdRp sequence has similarity to the RdRp of viruses from the family *Tombusviridae *and genus *Umbravirus*, the remaining SOG sequence has no detectable similarity to any other known sequence. A Bayesian maximum likelihood tree based on alignments of the SOG RdRp with the available *Umbravirus *sequences and representative members of the *Tombusviridae *indicates that the SOG genome forms a well supported clade (Bayesian clade support value of 100) with the single member of the genus *Avenavirus*, OCSV (Figure 5). Additionally, the presence of an amber stop codon (nt position 1230–1232) at the end of ORF 1 of the SOG genome (Figure 2C), resembles the in-frame termination codon characteristic of the replicase gene of viruses in 7 of the 8 genera of the *Tombusviridae *[24]. This division of the replicase of the *Tombusviridae *by a termination codon is thought to be part of a translational read though gene expression strategy [24]. Other similarities to the *Tombusviridae *include a similar genome size, the absence of an obvious helicase motif and the 5' proximal relative position of the RdRp within the genome [22]. However, unlike viruses in the *Tombusviridae*, there is no recognizable sequence for conserved movement or capsid proteins in the SOG genome. The absence of a recognizable movement protein could indicate the SOG virus does not infect a higher plant. Our inability to identify structural genes may indicate that, like the umbraviruses, the SOG virus does not encode capsid proteins. However, it is also possible that movement or structural proteins encoded in the SOG genome have no sequence similarity to those currently in the NCBI database.

## Conclusion

Our analyses suggest that a persistent, widespread and possibly dominant population of novel polycistronic picorna-like viruses is an important component of the RNA virioplankton in coastal waters. Nevertheless, as exemplified by the SOG genome from the Strait of Georgia site, other marine RNA virus assemblages appear to contain viruses whose detectable sequence similarity with established groups of viruses is limited to only the most conserved genes (i.e. RdRp). The novelty of JP-A, JP-B and SOG, as revealed by sequence analyses and genome characterization, suggests that most of the diversity in the marine RNA virus community remains uncharacterized. Furthermore, these results raise the hypothesis that the genomes of these marine RNA viruses that we propose to infect single-celled eukaryotes may be more similar to the ancestral RNA viruses that gave rise to those that infect higher organisms.

## Methods

### Station descriptions

The shotgun libraries were constructed from seawater samples collected from two stations, JP (Jericho Pier), a site in English Bay adjacent to the city of Vancouver, British Columbia and SOG (Strait of Georgia), located in the central Strait of Georgia next to Powell River, B.C. (Figure 1).

The locations of the stations where one or both of the JP genomes were detected are shown in Figure 2. Details for each station are listed in Table 2. In summary, samples were collected from sites throughout the Strait of Georgia, including repeated sampling from the JP site during different seasons, and from the West coast of Vancouver Island in Barkley Sound.

### Virus concentration method

Concentrated virus communities were produced as described by Suttle et al. [25]. Twenty to sixty litres of seawater from each station were filtered through glass fibre (nominal pore size 1.2 μm) and then 0.45 μm pore-size Durapore polyvinylidene fluoride (PVDF) membranes (Millipore, Cambridge, Canada), to remove particulates larger than most viruses. This filtrate was subsequently concentrated approximately 200 fold through a Tangential Flow Filter cartridge (Millipore) with a 30 kDa molecular cut-off, essentially concentrating the 2 to 450 nm size fraction of seawater. Remaining bacteria were removed by filtering the concentrate two times through a 0.22 μm Durapore PVDF membrane (Millipore). Virus-sized particles in each VC were pelleted via ultracentrifugation (5 h at 113 000 × g at 4°C). Pellets were resuspended overnight at 4°C in sterile 50 μM Tris chloride (pH 7.8).

### Whole genome library construction

A detailed description of the whole genome shotgun library construction protocol can be found in Culley et al. [10]. Briefly, before extraction, concentrated viral lysates were treated with RNase (Roche, Mississauga, Canada) and then extracted with a QIAamp Minelute Virus Spin Kit (Qiagen, Mississauga, Canada) according to the manufacturer's instructions. An aliquot of each extract was used in a PCR reaction with universal 16S primers to ensure samples were free of bacteria. To isolate the RNA fraction, samples were treated with DNase 1 (Invitrogen, Burlington, Canada) and used as templates for reverse transcription with random hexamer primers. Double-stranded (ds) cDNA fragments were synthesized from single stranded DNA with Superscript III reverse transcriptase (Invitrogen) using nick translational replacement of genomic RNA [26]. After degradation of overhanging ends with T4 DNA polymerase (Invitrogen), adapters were attached to the blunted products with T4 DNA ligase (Invitrogen). Subsequently, excess reagents were removed and cDNA products were separated by size with a Sephacryl column (Invitrogen). To increase the amount of product for cloning, size fractions greater than 600 bp were amplified with primers targeting the adapters. Products from each PCR reaction were purified and cloned with the TOPO TA Cloning system (Invitrogen). Clones were screened for inserts by PCR with vector-specific primers. Insert PCR products greater than 600 bp were purified and sequenced at the University of British Columbia's Nucleic Acid and Protein Service Facility (Vancouver, Canada). Sequence fragments were assembled into overlapping segments using Sequencher v 4.5 (Gene Codes, Ann Arbor, U.S.A.) based on a minimum match % of 98 and a minimum bp overlap of 20. Sequences were compared against the NCBI database with tBLASTx [12]. A sequence was considered significantly similar if BLAST *E *values were < 0.001. The details for viruses used in phylogenetic analyses are listed in additional file 2. Virus protein sequences were aligned using CLUSTAL X v 1.83 with the Gonnet series protein matrix [27]. Alignments were transformed into likelihood distances with Mr. Bayes v3.1.1 [21] and 250,000 generations. Neighbor-joining trees were constructed with PAUP v4.0 [28], and bootstrap values calculated based on percentages of 10,000 replicates.

### 5' and 3' RACE

The 5' and 3' ends of the environmental viral genomes were cloned using the 5' and 3' RACE systems (Invitrogen) according to manufacturer's instructions. The 3' RACE with the SOG genome required the addition of a poly (A) tract with poly (A) polymerase (Invitrogen) according to manufacturer directions before cDNA synthesis. cDNA was synthesized directly from extracted viral RNA from the appropriate library. Three clones of each 5' and 3' end were sequenced.

### PCR

#### Closing gaps in the assembly

PCR with primers targeting specific regions of the two JP environmental genomes were used to verify the genome assembly, increase sequencing coverage and reconfirm the presence of notable genome features. The template for these reactions was the amplified and purified PCR product from the JP and SOG shotgun libraries. Additional file 1 lists the sequence and genome position of primers used. The standard PCR conditions were reactions with 1 U of Platinum *Taq *DNA polymerase (Invitrogen) in 1× Platinum *Taq *buffer, 1.5 mM MgCl_2_, 0.2 mM of each dNTP, and 0.2 μM of each primer (see Additional file 1), in a final volume of 50 μl. Thermocycler conditions were, activation of the enzyme at 94°C for 1 min 15 s, followed by 30 cycles of denaturation at 94°C for 45 s, annealing at 50°C for 45s and extension at 72°C for 1 minute. The reaction was terminated after a final extension stage of 5 min at 72°C. PCR products were purified with a PCR Minelute cleanup kit (Qiagen) and sequenced directly with both primers.

#### Environmental screening

To assess the temporal and geographic distribution of the JP genomes, extracted RNA from viral concentrates were screened with Superscript III One-step RT-PCR System with Platinum *Taq *DNA Polymerase (Invitrogen) with primers JP-A 5 and 6 and JP-B 6 and 7 (see Additional file 1). The template for the reactions was DNase 1 treated viral RNA, extracted with a QIAamp Minelute Virus Spin Kit (Qiagen) according to the manufacturer's instructions. Each reaction consisted of RNA template, 1× reaction mix, 0.2 μM of each primer, 1 μl RT/Platinum *Taq *mix in a volume of 50 μl. Reactions were incubated 30 min at 50°C, then immediately heated to 94°C for 45 s, followed by 35 cycles of denaturation at 94°C for 15 s, annealing at 50°C for 30 s and extension at 68°C for 1 min. After a final extension step at 68°C for 5 min, RT-PCR products were analyzed by agarose gel electrophoresis. Products were sequenced to verify the correct target had been amplified.

## Competing interests

The author(s) declare that they have no competing interests.

## Authors' contributions

AC contributed to the design of the study, performed the lab work, analyzed the data and drafted the manuscript. AL contributed to the design of the study, analyzed the data and helped prepare the manuscript. CS was involved in the conceptualization and design of the research and in manuscript preparation. AC, AL and CS have read and approved this manuscript.

## Supplementary Material

Additional file 1 PCR primers used to complete the three genome sequences. The table provides detailed information about the primers used to complete the three viral genome sequences. Click here for file

Additional file 2 Virus sequence details. Organized by taxonomic group, the table provides the full name, acronym and NCBI accession number for the viruses used in phylogenetic analyses. Click here for file
