# Supplementary material for: The complete genomes of three viruses assembled from shotgun libraries of marine RNA virus communities
Source: Virol J. 2007 Jul 6;4:69. doi: 10.1186/1743-422X-4-69 (PMC1948888; doi:10.1186/1743-422X-4-69)
Supplement: Additional file 1 — PCR primers used to complete the three genome sequences. The table provides detailed information about the primers used to complete the three viral genome sequences. [file 1743-422X-4-69-S1.doc]

## Supplementary Table 1 - PCR primers used to complete the three genome sequences.

| Genome | Primer | Sequence (5’-3’) | Location (bp) | Strand primer is based on |
| --- | --- | --- | --- | --- |
| JP-A | JP-A-1 | TTATTGCTAAGGCTGAAAGTCT | 2596-2617 | + |
|  | JP-A-2 | ATCCATTTTCTACCAACTTCAC | 3467-3484 | - |
|  | JP-A-3 | TCGTCGGGAAGATGGC | 3764-3779 | + |
|  | JP-A-4 | GAAGCCTGCCACATCAAT | 4285-4300 | - |
|  | **JP-A-5** | **ATGGTGGCAGTATGGTCG** | **5552-5569** | **+** |
|  | **JP-A-6** | **CACTGGTATTCTTTGATTTTGAT** | **6165-6185** | **-** |
|  | JP-A-7 | TTGTGGATGATTCTGAACTTG | 6881-6901 | + |
|  | JP-A-8 | AAAATCGTCTCCAGCAGC | 7863-7878 | - |
|  | JP-A-9 | TTGCTCCTTATGCTCCTCA | 7943-7961 | + |
|  | JP-A-10 | GAAGGTTCTGGTGTTTATTTGTA | 8881-8901 | - |
| JP-B | JP-B-1 | CAATCATACCCCTGAGTTTAGA | 213-234 | + |
|  | JP-B-2 | AGTCTCAACAACACCCAAGC | 1058-1077 | - |
|  | JP-B-3 | CCCGATTTTCTGTATGTTTTAG | 1397-1418 | + |
|  | JP-B-4 | ACCAACGACCAACTTAGCC | 2076-2094 | - |
|  | JP-B-5 | GCGAAATGAAAAGGAGAAG | 2646-2664 | + |
|  | **JP-B-6** | **CGCTCTCGGACATAACAAA** | **3150-3168** | **-** |
|  | **JP-B-7** | **CCGTTTTCCGTTACATTGA** | **3666-3684** | **+** |
|  | JP-B-8 | TTTTACCAACCTTAGCCTTCT | 4240-4260 | - |
|  | JP-B-9 | GCTTCTTACTAAATCAATCCTTCTA | 5521-5545 | + |
|  | JP-B-10 | GCTAAAGTACAACCATAGAAAAATG | 6416-6440 | - |
| SOG | SOG-1 | ATACTTCTTCCCGCATCAG | 378-398 | + |
|  | SOG-2 | TCCTTGGAATCGCTTGTTGT | 771-790 | - |
|  | SOG-3 | CGTCGGGTCGTCTAAAAC | 1021-1040 | + |
|  | SOG-4 | CAGGCTTCTGAGGTGTGG | 1464-1481 | - |
|  | SOG-5 | GACTCCAACACAACAAATCG | 2716-2737 | + |
|  | SOG-6 | GAGACAGGACAAGCGTTATG | 3160-3179 | - |

Primers JP-A 5 and 6 and JP-B 6 and 7 (shown in bold) were used in the environmental survey.
