# Supplementary material for: The complete genomes of three viruses assembled from shotgun libraries of marine RNA virus communities
Source: Virol J. 2007 Jul 6;4:69. doi: 10.1186/1743-422X-4-69 (PMC1948888; doi:10.1186/1743-422X-4-69)
Supplement: Additional file 2 — Virus sequence details. Organized by taxonomic group, the table provides the full name, acronym and NCBI accession number for the viruses used in phylogenetic analyses. [file 1743-422X-4-69-S2.doc]

***Supplementary Table 2 – Virus sequence details***

| Virus Group | Virus Acronym | Full Name | NCBI Accession Number |
| --- | --- | --- | --- |
| Cheravirus | ALSV | Apple latent spherical virus | NC_003787 |
|  | CRLV | Cherry rasp leaf virus | NC_006271 |
|  |  |  |  |
| *Comoviridae* | BBWV1 | Broad bean wilt virus 1 | NC_005289 |
|  | CPMV | Cowpea mosaic virus | NC_003549 |
|  | TRSV | Tobacco ringspot virus | NC_005097 |
|  |  |  |  |
| *Dicistroviridae* | ABPV | Acute bee paralysis virus | NC_002548 |
|  | BQCV | Black queen cell virus | NC_003784 |
|  | CrPV | Cricket paralysis virus | NC_003924 |
|  | DCV | Drosophila C virus | NC_001834 |
|  | HiPV | Himetobi P virus | NC_003782 |
|  | PSIV | Plautia stali intestine virus | NC_003779 |
|  | TSV | Taura syndrome virus | NC_003005 |
|  | TrV | Triatoma virus | NC_003783 |
|  |  |  |  |
| Iflavirus | DWV | Deformed wing virus | NC_004830 |
|  | IFV | Infectious flacherie virus | NC_003781 |
|  | KV | Kakugo virus | NC_005876 |
|  | PnPV | *Perina nuda* picorna-like virus | NC_003113 |
|  | SbV | Sacbrood virus | NC_002066 |
|  | VDV | Varroa destructor virus | NC_006494 |
|  |  |  |  |
| *Marnaviridae* | HaRNAV | *Heterosigma akashiwo* RNA virus | NC_005281 |
|  |  |  |  |
| *Picornaviridae* | AiV | Aichi virus | NC_001918 |
|  | EMCV | Encephalomyocarditis virus | NC_001479 |
|  | ERBV | Equine rhinitis B virus 1 | NC_003983 |
|  | FMDV | Foot-and-mouth disease virus A | NC_011450 |
|  | HAV | Hepatitis A virus | NC_001489 |
|  | HPeV | Human parechovirus | NC_001897 |
|  | HRV | Human rhinovirus 14 | NC_001490 |
|  | PV | Poliovirus | NC_002058 |
|  | PTV | Porcine teschovirus 1 | NC_003985 |
|  |  |  |  |
| Sadwavirus | SDV | Satsuma dwarf virus | NC_003785 |
|  | NIMV | Navel orange infectious mottling virus | AB022887 |
|  |  |  |  |
| *Sequiviridae* | MCDV | Maize chlorotic dwarf virus | NC_003626 |
|  | PYFV | Parsnip yellow fleck virus | NC_003628 |
|  | RTSV | Rice tungro spherical virus | NC_001632 |
| *Tombusviridae* | CaRMV | Carnation mottle virus | NC_001265 |
|  | CRSV | Carnation ringspot virus | NC_003530 |
|  | MCMV | Maize chlorotic mottle virus | NC_003627 |
|  | OCSV | Oat chlorotic stunt virus | NC_003633 |
|  | PMV | Panicum mosaic virus | NC_002598 |
|  | PoLV | Pothos latent virus | NC_000939 |
|  | TBSV | Tomato bushy stunt virus | NC_001554 |
|  | TNV-A | Tobacco necrosis virus A | NC_001777 |
|  |  |  |  |
| Umbravirus | CMoMV | Carrot mottle mimic virus | NC_001726 |
|  | GRV | Groundnut rosette virus | NC_003603 |
|  | PEMV-2 | Pea enation mosaic virus-2 | NC_003853 |
|  | TBTV | Tobacco bushy top virus | NC_004366 |
|  |  |  |  |
| Unclassified | RsRNAV | *Rhizosolenia setigera* RNA virus | AB243297 |
|  | SssRNAV | *Schizochytrium* single-stranded RNA virus | NC_007522 |
